# Cervical Cancer Screening in the United States Military Health System During the COVID-19 Pandemic

**DOI:** 10.7759/cureus.74510

**Published:** 2024-11-26

**Authors:** S. Ahmed Hussain, Michael Dore, Kelsey Van Bokkem, Julie R Whittington

**Affiliations:** 1 Gynecologic Surgery and Obstetrics, Naval Medical Center Portsmouth, Portsmouth, USA; 2 General Internal Medicine, Duke University School of Medicine, Durham, USA

**Keywords:** cervical cancer, early detection of cancer, military medicine, pap smear, uterine cervical neoplasm

## Abstract

Introduction

During the COVID-19 pandemic, healthcare systems implemented restrictions on in-person appointments to mitigate viral spread among healthcare workers and patients. This study assesses changes in cervical cancer screening (CCS) rates within the United States Military Health System (MHS) during this period. To date, no such data have been reported on COVID-19’s effect on CCS within the MHS.

Methods

This retrospective cohort study compares CCS rates from the pandemic period of February 1, 2020, to February 28, 2022, to a pre-pandemic cohort spanning January 1, 2013, to January 31, 2020. Screening rates were analyzed using interrupted time series and regression methods.

Results

Results indicate a statistically significant decline in adequately screened patients, dropping from 77.9% (684,923 of 879,091 eligible patients) in January 2013 to 70.0% (457,109 of 652,507 eligible patients) in February 2021 (p<0.05). A statistically significant drop was also noted when comparing February 2020 (76.5%, 583,941 of 763,692 eligible patients) to February 2021 (70.0%, 457,109 of 652,507 eligible patients; p<0.05) and to February 2022 (72.3%, 496,100 of 686,029 eligible patients; p<0.05). The average pre-pandemic CCS rate of 75.5% significantly differed from the pandemic period's average of 73.3% (p<0.00001), representing 17,452 patients with inadequate screening during the pandemic.

Conclusion

This study highlights a substantial reduction in CCS within the MHS during the COVID-19 pandemic, aligning with national trends in cancer screening. It underscores the need for sustained healthcare access during crises and emphasizes the importance of planning to uphold essential preventative services. Future research should explore strategies to mitigate pandemic-related disruptions in cancer screening and their long-term implications on public health.

## Introduction

As the coronavirus disease 2019 (COVID-19) lockdowns began in the spring of 2020, many healthcare systems restricted their operations for the health and safety of their providers and their patients. Health systems redirected resources by reducing the availability of in-person appointments to slow the progression of the virus and reduce exposure to healthcare workers and patients alike. As a result, many preventative health screenings were restricted in their availability, causing a decrease in screening rates for various cancers [1-3].

Screening for breast, colorectal, and prostate cancers declined markedly in 2020, with an estimated COVID-19-related screening deficit for these three cancers affecting 9.4 million patients [1]. However, these screening declines varied by geographic region and socioeconomic status. The largest declines occurred in the Northeastern United States, and the smallest declines occurred in the West and South [1]. Additionally, cancer screenings requiring a procedure, such as mammography or colonoscopy, were the most significantly affected [1].

Preventative health screenings are crucial for early detection and treatment of cancers, and any disruptions can have long-term consequences on patient outcomes. This makes understanding the impact of COVID-19 on such screenings an important area of research. The pandemic caused a shift in healthcare priorities, with many resources being diverted to manage COVID-19 cases, leaving other essential services, such as cancer screenings, understaffed.

Cervical cancer screening is an essential preventative measure that can significantly reduce the incidence and mortality of cervical cancer [4]. The screening process involves testing, such as cervical cytology (Pap smear) and human papillomavirus (HPV) testing, which can detect precancerous changes and HPV infections that may lead to cervical cancer [4]. Regular screening and early detection are key components in reducing cervical cancer rates and improving survival outcomes [5].

Our study seeks to evaluate the change in the rate of cervical cancer screenings within the United States Military Health System (MHS) during the response to COVID-19. The MHS is geographically diverse, with facilities throughout the United States and in several international locations. While the socioeconomic status of its patient population also varies widely, the population benefits from healthcare services without cost at the point of care to the patient. This unique characteristic of the MHS provides an interesting context to study the impact of COVID-19 on cervical cancer screening, as it eliminates the financial barriers that might affect screening rates in the general population.

As appointment availability decreased and military physicians were mobilized with COVID-19 missions, the resources available for cervical cancer screening were diminished. Studies in the civilian population demonstrated a decrease of 5-35% in cervical cancer screening in 2019-2020 compared to pre-pandemic levels [2]. No studies have been performed to evaluate the effect of the COVID-19 pandemic on cervical cancer screening in the MHS. This study aims to provide insights into how the MHS managed cervical cancer screening during the pandemic.

## Materials and methods

This is a retrospective cohort study that compares the percentage of patients with up-to-date cervical cancer screening (cervical cancer screening) during the COVID-19 pandemic period of February 1, 2020, to February 28, 2022, to a historic pre-pandemic cohort from January 1, 2013, to January 31, 2020. Data were obtained from CarePoint and included all 24- to 64-year-old women continuously enrolled in TRICARE Prime during the study period. Women were considered to have adequate cervical cancer screening if they met at least one of the following three criteria: Cervical cytology in the past three years, HPV testing in the past five years (where the woman was over 30 years old), and cervical cytology and HPV co-testing in the past five years (where the woman was over 30 years old at the time of the co-test) [6].

The number of patients who had adequate cervical cancer screening was compared to the total patient population meeting eligibility criteria for cervical cancer screening as well as to the expected percentage of adequately screened patients as calculated by a regression analysis of the pre-pandemic screening trend. Statistical analysis was performed using an interrupted time series analysis.

The study population included women aged 24-64 years who were continuously enrolled in TRICARE Prime, a health insurance program offered to military personnel and their dependents. This population is unique because it includes individuals from diverse geographic regions and socioeconomic backgrounds, but all have access to healthcare services without cost at the point of care. This aspect of the MHS removes financial barriers to screening, providing a clear view of how operational changes, rather than economic factors, affected screening rates.

Patients were excluded if they were 65 years or over, if they had undergone hysterectomy, if they had a diagnosis of cervical cancer in their medical record, and if they had not been continuously enrolled in TRICARE Prime during the study period.

Data collection involved reviewing medical records from the CarePoint system, which tracks healthcare utilization and outcomes for TRICARE enrollees. The study focused on determining the proportion of women who were up-to-date with their cervical cancer screening based on the criteria mentioned above. These criteria align with the guidelines provided by the U.S. Preventive Services Task Force (USPSTF) and other major medical organizations, ensuring that the screening practices studied are consistent with national standards.

Interrupted time series analysis was chosen as the statistical method to assess the impact of the COVID-19 pandemic on cervical cancer screening rates. This method is well-suited for evaluating the effects of interventions or disruptions over time, allowing for a comparison of trends before and after the onset of the pandemic. By comparing the observed screening rates during the pandemic to the expected rates based on pre-pandemic trends, we can quantify the impact of the pandemic on screening behaviors within the MHS. P-values were generated using chi-square tests.

This study used an anonymized database and was thus deemed exempt by the Institutional Review Board.

## Results

**Figure 1:**
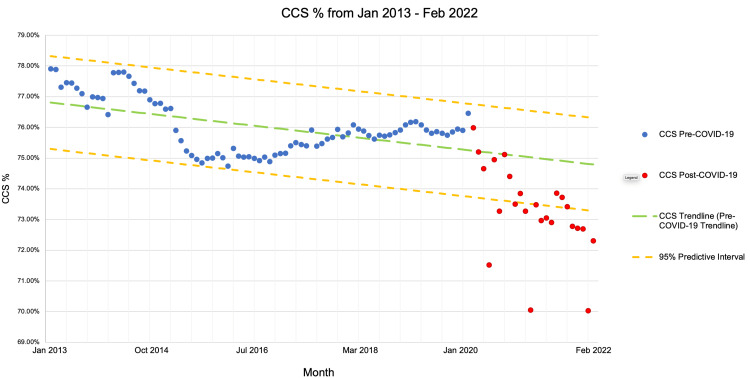
CCS rates from January 2013 to February 2022 CCS, cervical cancer screening

Prior to the COVID-19 pandemic, the percentage of TRICARE Prime enrollees with adequate cervical cancer screening declined from 77.9% in January 2013 to 76.5% in February 2020 (Table 1), a difference which was not statistically significant (p>0.05). Cervical cancer screening declined from pre-pandemic levels to a nadir of 70.0% in February 2021, with a slight recovery over the following year to 72.3% in February 2022 (Table 2, Figure 1).

**Table 1:** Cervical cancer screening rates by month (January 2013 to February 2020)

	Adequately Screened (N)	Eligible (N)	Screened (%)
January 2013	684,923	879,091	77.91%
February 2013	683,403	877,431	77.89%
March 2013	649,421	840,057	77.31%
April 2013	678,199	875,507	77.46%
May 2013	731,407	944,314	77.45%
June 2013	726,747	940,468	77.28%
July 2013	720,235	934,198	77.10%
August 2013	717,166	935,573	76.66%
September 2013	709,195	920,996	77.00%
October 2013	709,000	920,999	76.98%
November 2013	704,198	915,311	76.94%
December 2013	691,172	904,404	76.42%
January 2014	702,367	902,973	77.78%
February 2014	694,321	892,541	77.79%
March 2014	684,114	879,225	77.81%
April 2014	686,366	883,747	77.67%
May 2014	683,365	882,492	77.44%
June 2014	680,997	882,073	77.20%
July 2014	688,979	892,581	77.19%
August 2014	680,371	884,737	76.90%
September 2014	677,152	881,914	76.78%
October 2014	677,759	882,621	76.79%
November 2014	675,215	881,539	76.60%
December 2014	673,733	879,346	76.62%
January 2015	669,780	882,281	75.91%
February 2015	660,547	874,125	75.57%
March 2015	658,305	874,954	75.24%
April 2015	652,403	868,853	75.09%
May 2015	658,521	878,531	74.96%
June 2015	655,609	875,896	74.85%
July 2015	657,350	876,626	74.99%
August 2015	650,352	867,101	75.00%
September 2015	652,008	867,631	75.15%
October 2015	648,200	864,174	75.01%
November 2015	646,873	865,441	74.74%
December 2015	649,743	862,587	75.32%
January 2016	637,271	848,891	75.07%
February 2016	642,768	856,585	75.04%
March 2016	643,288	857,104	75.05%
April 2016	641,296	855,219	74.99%
May 2016	638,279	851,956	74.92%
June 2016	640,727	853,815	75.04%
July 2016	629,561	840,662	74.89%
August 2016	636,164	847,125	75.10%
September 2016	630,219	838,586	75.15%
October 2016	626,449	833,495	75.16%
November 2016	636,055	843,613	75.40%
December 2016	636,830	843,345	75.51%
January 2017	632,915	838,898	75.45%
February 2017	629,995	835,525	75.40%
March 2017	635,559	837,174	75.92%
April 2017	629,063	834,411	75.39%
May 2017	630,898	835,803	75.48%
June 2017	626,524	828,476	75.62%
July 2017	621,932	821,739	75.68%
August 2017	629,951	829,498	75.94%
September 2017	614,945	812,346	75.70%
October 2017	602,835	795,086	75.82%
November 2017	608,198	799,312	76.09%
January 2018	603,636	794,782	75.95%
February 2018	598,099	788,156	75.89%
March 2018	597,045	788,333	75.74%
April 2018	595,863	787,987	75.62%
May 2018	596,949	788,054	75.75%
June 2018	585,229	772,919	75.72%
July 2018	592,202	781,700	75.76%
September 2018	591,623	780,227	75.83%
October 2018	588,875	775,691	75.92%
November 2018	589,276	774,453	76.09%
December 2018	592,002	777,172	76.17%
January 2019	588,700	772,707	76.19%
February 2019	589,551	774,788	76.09%
March 2019	584,770	770,282	75.92%
June 2019	586,869	774,106	75.81%
July 2019	586,521	773,129	75.86%
August 2019	585,372	772,122	75.81%
September 2019	575,749	760,032	75.75%
October 2019	576,534	760,062	75.85%
November 2019	574,949	757,016	75.95%
December 2019	571,486	752,864	75.91%
February 2020	583,941	763,692	76.46%

**Table 2:** Cervical cancer screening rates by month (February 2020 to February 2022)

	Adequately Screened (N)	Eligible (N)	Screened (%)
February 2020	583,941	763,692	76.46%
March 2020	577,256	759,599	75.99%
April 2020	574,766	764,351	75.20%
May 2020	572,129	766,300	74.66%
June 2020	628,886	879,320	71.52%
July 2020	534,565	713,210	74.95%
August 2020	481,530	657,180	73.27%
September 2020	563,632	750,324	75.12%
October 2020	554,861	745,694	74.41%
November 2020	543,929	739,942	73.51%
December 2020	565,298	765,506	73.85%
January 2021	547,983	747,878	73.27%
February 2021	457,109	652,507	70.05%
March 2021	546,074	743,185	73.48%
April 2021	500,391	685,751	72.97%
May 2021	500,939	685,765	73.05%
June 2021	501,303	687,568	72.91%
July 2021	551,702	746,924	73.86%
August 2021	551,626	748,120	73.73%
September 2021	531,329	723,712	73.42%
October 2021	533,367	732,890	72.78%
November 2021	534,295	734,691	72.72%
December 2021	533,390	733,662	72.70%
January 2022	419,864	599,569	70.03%
February 2022	496,100	686,029	72.31%

The predicted screening rates associated with these time periods based on modeling were 75.0% (95% CI 74.86-75.19%) for February 2021 and 74.8% (95% CI 74.62-74.96%) for February 2022. Both predictions differed from their respective observed values in a statistically significant manner (p<0.05).

**Figure 2:**
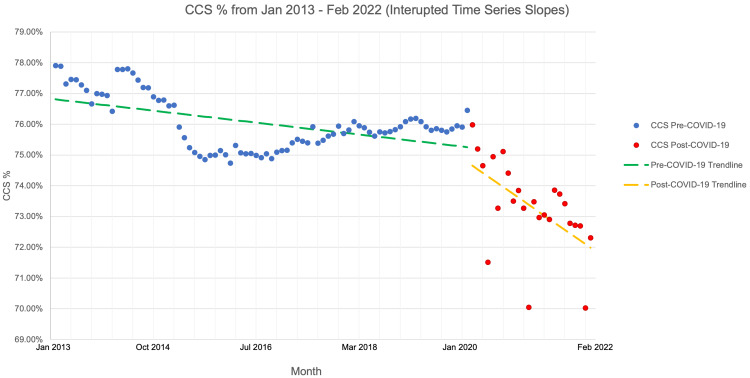
CCS rates from January 2013 to February 2022 (interrupted time series slopes) CCS, cervical cancer screening

An interrupted time series analysis was then performed with two independent regression analyses. One was performed for the pre-pandemic period of January 1, 2013, to January 31, 2020, and another for the pandemic period of February 1, 2020, to February 28, 2022 (Figure 2).

There was a statistically significant decrease in the percentage of adequately screened patients in January 2013 (77.9%) compared to the percentage of such patients in February 2021 (70.0%, p<0.05). This difference was also statistically significant when comparing February 2020 (76.5%) to February 2021 (p<0.05). Similarly, there was a statistically significant decrease in the percentage of adequately screened patients in February 2020 (76.5%) when compared to the percentage of such patients in February 2021 (p<0.05) as well as with February 2022 (72.3%, p<0.05).

Next, the average percentage of adequately screened patients in the pre-pandemic period (75.5%) was compared to the average percentage of adequately screened patients in the pandemic period (73.3%). Using a chi-square analysis, this difference of 2.2% was found to be statistically significant with a p-value of <0.00001. This difference corresponds to an excess of 17,452 patients with inadequate screening in the pandemic period compared to the pre-pandemic timeframe.

## Discussion

The results indicate a clear and significant impact of the COVID-19 pandemic on cervical cancer screening rates within the MHS. The decline in screening rates during the pandemic period suggests that the operational changes and resource reallocations within the MHS had a tangible effect on the delivery of preventative healthcare services. The nadir in screening rates observed in February 2021 coincides with the peak periods of COVID-19 case surges, reflecting the heightened strain on healthcare systems during these times.

The slight recovery in screening rates by February 2022 suggests some resilience and adaptation within the MHS, but the rates had not returned to pre-pandemic levels. This ongoing deficit highlights the need for targeted interventions to address the backlog of missed screenings and to ensure that screening services can continue effectively in the face of ongoing public health challenges.

This study demonstrates that the rate of cervical cancer screening in the MHS from February 1, 2020, to February 28, 2022, was significantly lower than that of the preceding seven years, resulting in an excess of 17,452 inadequately screened patients and potentially missing 959 abnormal pap smears. Using the previously reported rate of 19.4% of these abnormal findings resulting in a diagnosis of cervical intraepithelial neoplasia (CIN) or cancer [7], this corresponds to a potential 186 cases of missed CIN or cervical malignancy.

This study additionally demonstrates that the decrease in cervical cancer screening during the pandemic period had not recovered to pre-pandemic levels as of February 2022, as there was still a statistically significant difference between the screening rates of that month and that of February 2020. The decrease in cervical cancer screening of 2.2% between the two study periods is modest but statistically significant. Similar studies in the civilian population report cervical cancer screening differences as low as 5% [2]. The smaller decrease reported here may be due to the relative ease of access that the military population has to primary healthcare services and due to the lack of cost at the point of care.

A strength of this study is the large and comprehensive dataset that reflects the cervical cancer screening rate within the entire MHS. This allows for a robust analysis of screening trends over a significant period. The consistency in healthcare access and the elimination of financial barriers within the MHS provide a unique opportunity to isolate the impact of operational disruptions on screening rates, independent of economic factors.

A limitation of this dataset is its reliance on accurate coding of the testing performed during every patient encounter. Inaccurate or incomplete coding could lead to misclassification of patients' screening status, potentially underestimating or overestimating the true screening rates. Another limitation is that cervical cancer screening is based on the current U.S. Preventive Services Task Force guidelines, which were updated in 2018 with the addition of HPV testing after it was approved by the FDA in 2014 [8]. Other institutions including the American College of Physicians and the American College of Obstetricians and Gynecologists updated their guidelines earlier in 2015 and 2016, respectively [9,10]. These additional guidelines may have changed individual practice patterns, introducing variability in screening practices over time.

Due to limitations with the MHS dataset, it was not possible to exclude patients with an existing diagnosis of cervical dysplasia as these patients. These patients would potentially be incorrectly considered to have adequate cervical cancer screening as American Society for Colposcopy and Cervical Pathology guidelines may require them to be screened with a smaller interval than three to five years. Thus, our study may underestimate the number of patients with inadequate screening.

Additionally, this study is correlative and retrospective in nature. Though the decrease in screening corresponds temporally with the arrival of COVID-19 to the United States and with the timing of Military Treatment Facilities’ implementation of pandemic protocols, this study is not designed to identify the underlying cause of that decrease. It must also be noted that servicemembers on international bases may have had different recommendations or restrictions depending on their host country. Therefore, no conclusions can be drawn regarding the causation of the decline in cervical cancer screening over this timeframe, as we cannot definitively report that the decrease in cervical cancer screening was due specifically to the pandemic or pandemic protocols. However, the data presented here are consistent with other trends in cancer screening that have been reported in the literature over the pandemic timeframe, such as decreased rates of breast, colorectal, and prostate cancer screening, as noted by Chen et al. [1].

Another area for future research involves exploring the long-term outcomes of the reduced screening rates observed during the pandemic. This may include studies on the stage at which cervical cancers are diagnosed post-pandemic and the associated outcomes compared to pre-pandemic diagnoses. Such research would provide valuable insights into the direct impact of screening delays on patient health and could help shape future public health strategies. Furthermore, additional studies may investigate the effectiveness of different interventions implemented to mitigate the impact of the pandemic on cancer screening rates, such as the use of telehealth services, patient education programs, and enhanced scheduling flexibility.

The findings of this study highlight the importance of maintaining essential healthcare services during public health crises. The COVID-19 pandemic has underscored the need for healthcare systems to be resilient and adaptable, ensuring that preventative services such as cancer screenings are not unduly compromised. Future planning and policy-making should consider the development of robust contingency plans that can sustain critical healthcare operations even during times of significant disruption.

COVID-19 continues with more than 300,000 new cases every month worldwide [11]. Though the lockdowns of the initial months of the pandemic are likely in the past, the public health consequences of this disease will take years, if not decades, to emerge. Its downstream effects will manifest in many areas of epidemiology, including rates of cancer and cancer mortality. It is essential that cancer screening during the pandemic be reported in order for proactive strategies to be implemented to minimize any effects on future cancer rates.

## Conclusions

The findings of this study underscore the critical need for healthcare systems to maintain essential services such as cancer screening during times of crisis. Strategies to achieve this could include the implementation of telehealth services, increased flexibility in scheduling, and ensuring that healthcare workers have the necessary resources and support to continue providing care. By learning from the challenges posed by the COVID-19 pandemic, healthcare systems can be better prepared to handle future emergencies without compromising the delivery of essential preventative services.

The study highlights the need for further research into the long-term impacts of reduced screening rates and the effectiveness of interventions designed to mitigate these impacts. Continued monitoring and analysis of screening rates and cancer outcomes will be crucial in understanding the full scope of the pandemic's effects on public health and in developing strategies to address any ongoing or future disruptions to healthcare services. This study is the first of its kind to analyze the difference in cervical cancer screening rates during the COVID-19 pandemic in the MHS. We report a statistically significant decrease in cervical cancer screening in the MHS that follows national trends in cervical, breast, colorectal, and prostate screening rates in the civilian population during the pandemic.
